# Analysis of the *Lotus japonicus* nuclear pore NUP107-160 subcomplex reveals pronounced structural plasticity and functional redundancy

**DOI:** 10.3389/fpls.2013.00552

**Published:** 2014-01-22

**Authors:** Andreas Binder, Martin Parniske

**Affiliations:** Faculty of Biology, Genetics, University of MunichMartinsried, Germany

**Keywords:** nuclear pore complex, nucleoporins, *Lotus japonicus*, NUP107-160 subcomplex, plant nucleus

## Abstract

Mutations in the *Lotus japonicus* nucleoporin genes, *NUP85, NUP133*, and *NENA (SEH1)*, lead to defects in plant-microbe symbiotic signaling. The homologous proteins in yeast and vertebrates are part of the conserved NUP84/NUP107-160 subcomplex, which is an essential component of the nuclear pore scaffold and has a pivotal role in nuclear pore complex (NPC) assembly. Loss and down-regulation of NUP84/NUP107-160 members has previously been correlated with a variety of growth and molecular defects, however, in *L. japonicus* only surprisingly specific phenotypes have been reported. We investigated whether Lotus *nup85, nup133*, and *nena* mutants exhibit general defects in NPC composition and distribution. Whole mount immunolocalization confirmed a typical nucleoporin-like localization for NUP133, which was unchanged in the *nup85-1* mutant. Severe NPC clustering and aberrations in the nuclear envelope have been reported for *Saccharomyces cerevisiae nup85* and *nup133* mutants. However, upon transmission electron microscopy analysis of *L. japonicus nup85*, *nup133* and *nena*, we detected only a slight reduction in the average distances between neighboring NPCs in *nup133*. Using quantitative immunodetection on protein-blots we observed that loss of individual nucleoporins affected the protein levels of other NUP107–160 complex members. Unlike the single mutants, *nup85/nup133* double mutants exhibited severe temperature dependent growth and developmental defects, suggesting that the loss of more than one NUP107–160 member affects basal functions of the NPC.

## Introduction

Eukaryotic cells are organized into membrane enclosed organelles, allowing for functional and spatial division of cellular processes. The nucleus is confined by a double membrane, the nuclear envelope, thus separating nuclear DNA from cytoplasm and uncoupling transcription and translation. Exchange of macromolecules in and out of the nucleus is mediated by nuclear pore complexes (NPCs), which are embedded into the nuclear membranes (Aitchison and Rout, 2012; Grossman et al., 2012). NPCs are large supramolecular assemblies, composed of about 30 proteins, termed nucleoporins (NUPs), which form stable subcomplexes. Each NUP is present in multiple copies due to the NPCs eight fold rotational symmetry and an imperfect symmetry between cytosolic and nucleoplasmic side (Alber et al., 2007; Hoelz et al., 2011). In addition to their role as gatekeepers in nucleocytoplasmic transport, NPCs and individual NUPs are involved in a large number of physiological processes, including kinetochore and spindle assembly, cell cycle control, regulation of gene expression, chromatin organization, DNA repair, and DNA replication (Capelson and Hetzer, 2009; Capelson et al., 2010; Strambio-De-Castillia et al., 2010; Wozniak et al., 2010; Van De Vosse et al., 2011, 2013; Bukata et al., 2013).

Nuclear pore components were mostly studied in yeast and vertebrates (Hoelz et al., 2011; Aitchison and Rout, 2012; Grossman et al., 2012), however relatively little is known about them in plants. A recent proteomics study in *A. thaliana* identified and characterized at least 29 nucleoporins, 22 of which were previously not annotated in plants (Tamura et al., 2010). Considering sequence similarity and domain organization, the plant proteins were more closely related to the vertebrate than to the yeast orthologs. For six vertebrate NUPs (NUP358, NUP188, NUP153, NUP45, NUP37, POM121), no homologs were found in *A. thaliana*, while one *A. thaliana* nucleoporin, NUP136/NUP1 was unique to plants, suggesting specific adaptations of the plant nuclear pore to particular aspects of plant cell biology (Tamura et al., 2010). The overall structure of the plant NPC resembles that of other eukaryotes (Fiserova et al., 2009), with an approximate diameter of 105 nm in *Nicotiana tobacum*, it is more similar to the vertebrate NPC (110–120 nm in *Xenopus*; Goldberg and Allen, 1996) than the smaller *Saccharomyces cerevisiae* NPC (95 nm; Kiseleva et al., 2004).

Forward and reverse genetic analyses implicated plant nucleoporins in plant microbe interactions, development, flowering, hormone- and cold stress signaling (Binder and Parniske, 2013). The majority of the genetically identified plant NUPs are homologous to members of the conserved nuclear pore NUP107–160 (in vertebrates) or NUP84 (in yeast) subcomplex. The complex forms a Y-shaped structure that is composed of at least seven core nucleoporins (NUP85, NUP96, NUP107, NUP133, NUP160, SEH1, SEC13; Belgareh et al., 2001; Lutzmann et al., 2002; Harel et al., 2003; Walther et al., 2003) and can contain up to three additional proteins (NUP37, NUP43, and ELYS) depending on the organism (Loïodice et al., 2004; Rasala et al., 2006). While the subcomplex has not been fully characterized in plants, it is likely conserved, as homologous genes of all core yeast NUP84 and vertebrate NUP107–160 members are present in *Arabidopsis* (Wiermer et al., 2012). Additionally, positive interaction of *L. japonicus* NUP85 and NENA (SEH1) in yeast-two-hybrid analysis is consistent with the arrangement of the homologous proteins in the *S. cerevisiae* NUP84 complex (Lutzmann et al., 2002; Groth et al., 2010). The subcomplex is the largest of the nuclear pore subunits and an essential structural component, required for nuclear pore assembly (Harel et al., 2003; Walther et al., 2003; Doucet et al., 2010). During mitosis it is recruited to kinetochores and contributes to normal kinetochore functions including spindle assembly (Belgareh et al., 2001; Orjalo et al., 2006; Zuccolo et al., 2007). Loss or depletion of individual NUP107–160/NUP84 NUPs is associated with different phenotypes. In *S. cerevisiae*, mutations in NUP84 members lead to temperature dependent growth defects, aberrant NPC biogenesis and distribution as well as accumulation of nuclear polyA RNA (Doye et al., 1994; Aitchison et al., 1995; Heath et al., 1995; Li et al., 1995; Pemberton et al., 1995; Siniossoglou et al., 1996; Fernandez-Martinez et al., 2012). In mice, NUP133 is dispensable for many essential NPC functions, however *nup133* mutants are disturbed in embryonic neural differentiation (Lupu et al., 2008). In contrast, loss of mouse NUP96 is lethal and low expression levels of NUP96 in heterozygous NUP96^+/−^ individuals affect interferon mediated immune responses (Faria et al., 2006). Mutations in *Arabidopsis thaliana NUP96, NUP160* and to a lesser extend in *SEH1* also lead to defects in basal and resistance (R)-gene mediated defense (Zhang and Li, 2005; Cheng et al., 2009; Wiermer et al., 2012). Additionally *NUP96* and *NUP160* are involved in auxin signaling (Parry et al., 2006) and *nup160* mutants are hypersensitive to ethylene (via the auxin pathway; Robles et al., 2012) and cold stress (Dong et al., 2006). Both *nup96* and *nup160* plants flower early and exhibit developmental defects (Parry et al., 2006). In *Lotus japonicus* three putative NUP107–160 complex nucleoporins, NUP85, NUP133, and the SEH1 homolog NENA are involved in the establishment of plant-microbe symbiosis. Mutations in the *NUP* genes disturb nuclear calcium spiking, an early physiological response in symbiotic signaling, and cause temperature dependent defects in arbuscular mycorrhiza (AM) and root nodule symbiosis (RNS) (Kistner et al., 2005; Kanamori et al., 2006; Saito et al., 2007; Groth et al., 2010). Furthermore, *L. japonicus nup85* mutants show defects in pollen tube growth and both *nup85* and *nup133* display a slight reduction in seed yield, which could not be observed for *nena*. However, no obvious additional growth or developmental phenotypes were reported for either of the mutants (Kanamori et al., 2006; Saito et al., 2007; Groth et al., 2010).

The lack of broad pleotropic defects at the plant level in the Lotus *nup* mutants prompted us to investigate potential changes in the nuclear organization with regard to NPC distribution and nuclear envelope structure. Based on the shared phenotypes of *nup85, nup133*, and *nena* mutants in plant-microbe symbiosis, we hypothesized that alterations in the NUP107–160 subcomplex could be responsible for the defects in symbiotic signaling. Investigation of the Lotus *nup* mutants for altered levels of NUP85 and NUP133 indicated that the loss of individual nucleoporins could lead to changes in the NUP107–160 subcomplex composition and thereby potentially result in the observed phenotypes.

The study of *nup85*/*nup133* double mutants revealed severe defects in growth and development, suggesting that the plant NPC cannot compensate effectively for the loss of more than one of these structural NUPs.

## Materials and methods

### Plant material and growth

*Lotus japonicus* ecotype B-129 Gifu was used as wild type. Seeds were scarified with sand paper and surface sterilized with 2% NaClO + 0.1% SDS. Seedlings were cultivated on 0.8% Bacto Agar (BD) at 18 or 24°C in 16 h light/8 h dark cycles.

### Crossing

*L. japonicus* flowers of the right stage (containing ripe anthers with unreleased pollen and a straight style) were selected and carefully emasculated by removing petals and anthers with forceps, without releasing the pollen (Jiang and Gresshoff, 1997). Pollen was harvested from the donor plant and released onto the thumb nail. The stigma of the emasculated flower was gently pressed into the pollen. The flower was covered with a plastic tube, which was stuffed with slightly moist cotton at the bottom for protection and to prevent cross-pollination.

### Genotyping

*L. japonicus nup85* and *nup133* crosses and their progeny were genotyped either by (derived) cleaved amplified polymorphism analysis (CAPs/dCAPs) or via the KASP system (LGC genomics). CAPs/dCAPs PCR was performed using 10–50 ng of genomic *L. japonicus* DNA as a template. For genotyping of NUP85 primers Nup85-1_dCAP_FW (TAAGGTGTTGTTTGTTTCTATTTTATAGCCATG) and Nup85-1_dCAP_Rv (GAAAATGAGAAACATACAACTCAATCC), for genotyping of NUP133 primers Nup133-1_CAPs_FW (TTCTGGCATTCATCGAACAG) and Nup133-1_CAPs_RV (TTTGACCAGCCAGATCCTTC) were used. PCR products were digested with restriction enzymes (NEB) NcoI-HF for NUP85 and RsaI for NUP133 and the products were separated on a 3% agarose gel. In case of NUP85, the wild type allele resulted in two bands of 30 and 177 bp and the *nup85-5* allele in a single band of 207 bp. In case of NUP133, the wild type allele showed two bands at 92 and 198 bp and the *nup133-1* allele a single band at 290 bp. KASP genotyping PCR was performed according to the manufacturer's instructions with specific primer sets for discrimination of wild type and *nup85/nup133* mutant alleles. KASP primers were designed by LGC genomics. Successful crosses and identified double mutants were confirmed by sequencing.

### Quantitative immunoblot

For production of peptide antibodies, peptides were selected that had no or low homology to other known proteins and contained predicted exposed epitopes, preferentially with several positively or negatively charged amino acids (R,H,K,D,E) to increase the potential binding avidity of the antibodies. Among the suitable sequences one peptide per protein was selected that contained few or no predicted phosphorylation and N-glycosylation sites. α-NUP85 peptide antibodies were raised in guinea-pigs against the peptide H_2_N—LHKYRDFKKSLQQVSGGK—CONH_2_ and α-NUP133 peptide antibodies were raised in rabbits against the peptide H_2_N—MHLPPEGGDSGQLEGNKGYPR—CONH_2_. Antibodies were produced by Pineda Antibody Service (Berlin). For total protein extraction two week old *L. japonicus* roots were ground in liquid nitrogen and taken up (100 mg/200 μl) in denaturating extraction buffer (10 mM EDTA, 50 mM Hepes, 150 mM NaCl, 10% Sucrose, 5 M Urea, 2 M Thiourea, 1.5% Triton-X 100, 1% SDS, 2 mM DTT, 1 mM PMSF, Sigma protease inhibitor cocktail P9599; Sigma-Aldrich). The solution was incubated for 1 h at 37°C. Proteins were separated on a polyacrylamide SDS gel and transferred for 3 h at 30 V and 1 h at 100 V onto a low-background Immobilon FL PVDF membrane (Millipore). Membranes were blocked in 5% skim milk in TBS (+0.1% Tween 20) and incubated overnight at 4°C in the primary antibodies and for 2 h in the secondary antibodies, both diluted in TBS (+3% skim milk and 0.1% Tween 20). Primary α-NUP85 (Guinea-Pig) antibody was used at a dilution of 1:2000, α-NUP133 (rabbit) at 1:5000 and α-Histone H3 (mouse; ab1791; abcam) at 1:500. Secondary α-guinea-PIG 680 LT (LI-COR) and secondary α-rabbit 680 LT (LI-COR) antibodies were diluted to 1:20000, secondary α-mouse 800 (Biomol) to 1:10000. Quantification and image acquisition of the secondary antibodies were performed with a LI-COR Odyssey Infrared imager using the Odyssey software.

### Whole-mount immunolocalization

Immunolocalization of *L. japonicus* root tissue was performed based on Sauer et al. (2006). *L. japonicus* plants were grown for 2 weeks on 0.8% water agar. Root tips (0.3–0.5 cm) were cut off with a razor blade, placed in freshly prepared fixative solution (4% Paraformaldehyde dissolved in 1 × PBS with 0.1% Triton X-100) and vacuum infiltrated for 1 h. Following removal of the fixative, the plant tissue was washed four times for 10 min with 1 × PBS. Root tips were placed on a small droplet of ddH_2_O on SuperFrost® Plus adhesive slides (Menzel-Gläser) and dried overnight at room temperature (RT). The plant tissue was rehydrated with a drop of 1 × PBS and placed in a humid chamber. 2% Driselase (D8037-1G; Sigma-Aldrich) was added for cell wall digestion and roots were incubated for 60 min at 37°C. After washing with 1 × PBS for six times, 3% IGEPAL CA-630 (Sigma-Aldrich) with 10% DMSO was added. These and successive washing steps were carried out in an excess volume of 1 × PBS in microscopy slide holders. For permeabilization, roots were incubated for 60 min at RT, then washed five more times with 1 × PBS. As blocking solution 3% albumin fraction V (BSA; Sigma-Aldrich) in 1 × PBS (100–200 μl) was added for 60 min at RT. After removal of the BSA solution, primary antibodies (α-NUP133; 1:500) were added in 3% BSA solution, in a sufficient amount to fully cover the roots. Slides were placed in a humid chamber and incubated for 240 min at 37°C, then overnight at 4°C. The samples were washed six times in 1 × PBS. Secondary antibody (Alexa Fluor® 488 α-rabbit; Life Technologies) in 3% BSA was added and roots were incubated for another 180 min at 37°C. Slides were again washed for five times in 1 × PBS. To visualize the nuclei 1 μg/ml 4′,6-Diamidino-2-phenylindole dihydrochloride (DAPI; Sigma-Aldrich) solution diluted in ddH_2_O was added for 10 min. Finally the root tips were washed three times with ddH_2_O to remove excess DAPI and salt. The samples were mounted in 0.25% 1,4-Diazabicyclo[2.2.2]octan (DABCO; Sigma-Aldrich), 90% glycerol in 1 × PBS and stored at 4°C in the dark.

Samples were analyzed by 3D structured illumination microscopy on a DeltaVision OMX (Applied precision) microscopic imaging system with a plan-apochromatic 100×, 1.4 NA, oil-immersion objective (Olympus). A 405 laser and a 488 laser were used to excite the DAPI stained DNA and Alexa® Fluor 488 labeled secondary antibodies, respectively. ImageJ software was used for image processing and generation of Z-stacks.

### Electron microscopy and NPC distance quantification

Root tips of 10 days-old *L. japonicus* Gifu wild type, *nup85-1*, *nup133-1*, and *nena-1* plants were fixed with 2.5% glutaraldehyde in fixation buffer (75 mM sodium cacodylate; 2 mM MgCl2; pH 7) overnight, then washed four times in fixation buffer and postfixed for 4 h with 1% osmium tetroxide in fixation buffer. After two additional washing steps with fixation buffer and distilled water, the root tips were dehydrated in an acetone dilution series (10, 20, 40, 60, 80, 100%, 20 min each). For staining the 20% acetone was supplemented with 1% uranyl acetate. The samples were embedded in Spurr's low viscosity resin by a gradual infiltration series (resin/acetone: 1/1, 2/1, 3/1, 4/1, 5/1, 4 h each, then 100% resin overnight) and polymerized at 63°C. Ultrathin sections of 50–70 nm were cut with a diamond knife and mounted on uncoated copper grids. The sections were poststained with aqueous lead citrate (100 mM, pH 13). Micrographes were taken with an EM 912 electron microscope (Zeiss) equipped with an integrated OMEGA energy filter operated in zero-loss mode. For each line approximately 10 individual root tips were analyzed. NPCs were quantified using Adobe Illustrator by measuring the distances between the centers of two adjacent nuclear pores.

Statistical tests were performed using R 2.15.1 software (R Core Team, 2013).

## Results

### Localization of *L. japonicus* NUP133 was unchanged in the *nup85* mutant

**Figure 1:**
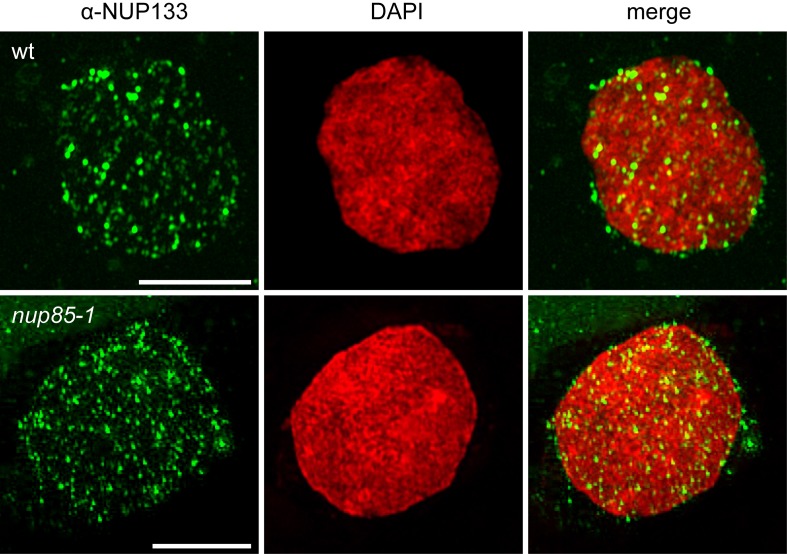
**Indirect immunolocalization of *L. japonicus* NUP133**. 3D structured illumination microscopy (Z-stack) detected a punctated NUP133 signal around the nucleus, corresponding to individual NPCs or clusters of nuclear pores. No change to NUP133 localization was observed in the *nup85-1* mutant. The α-NUP133 primary antibody was visualized with an Alexa Fluor® 488 labeled secondary antibody (green). DNA was stained with DAPI (red). wt, wild type. Scale bars = 5 μm.

Nuclear envelope localization of *L. japonicus* NUP133 was corroborated by whole-mount immunolocalization with a custom α-NUP133 antibody. Antibody specificity was confirmed by the absence of the nuclear signal in the *nup133-1* mutant (Table 1; Supplementary Figure 1). Using super-resolution 3D structured illumination microcopy (3D-SIM), NUP133 revealed a dotted localization pattern in the circumference of the nuclear rim, in accordance with its predicted function as a nucleoporin (Figure 1). Individual dots likely corresponded to single NPCs or NPC clusters. The observed localization was unchanged in *nup85* nuclei, suggesting that the overall NPC distribution was not affected in the mutant.

**Table 1:** **Analyzed *L. japonicus* nucleoporin mutant lines**.

**Line**	**Genomic mutation**	**Effect**	**References**
*nup85-1*	G_4992_ to A	W_306_ to STOP	Kistner et al., 2005; Saito et al., 2007
*nup133-1*	A_1196_-T_1205_ deletion	Frame shift	Kanamori et al., 2006
*nena-1*	C_400_T	Q_97_ to STOP	Groth et al., 2010

### *nup85*, *nup133*, and *nena* mutants did not exhibit clustering of NPCs or aberrations in the nuclear envelope

**Figure 2:**
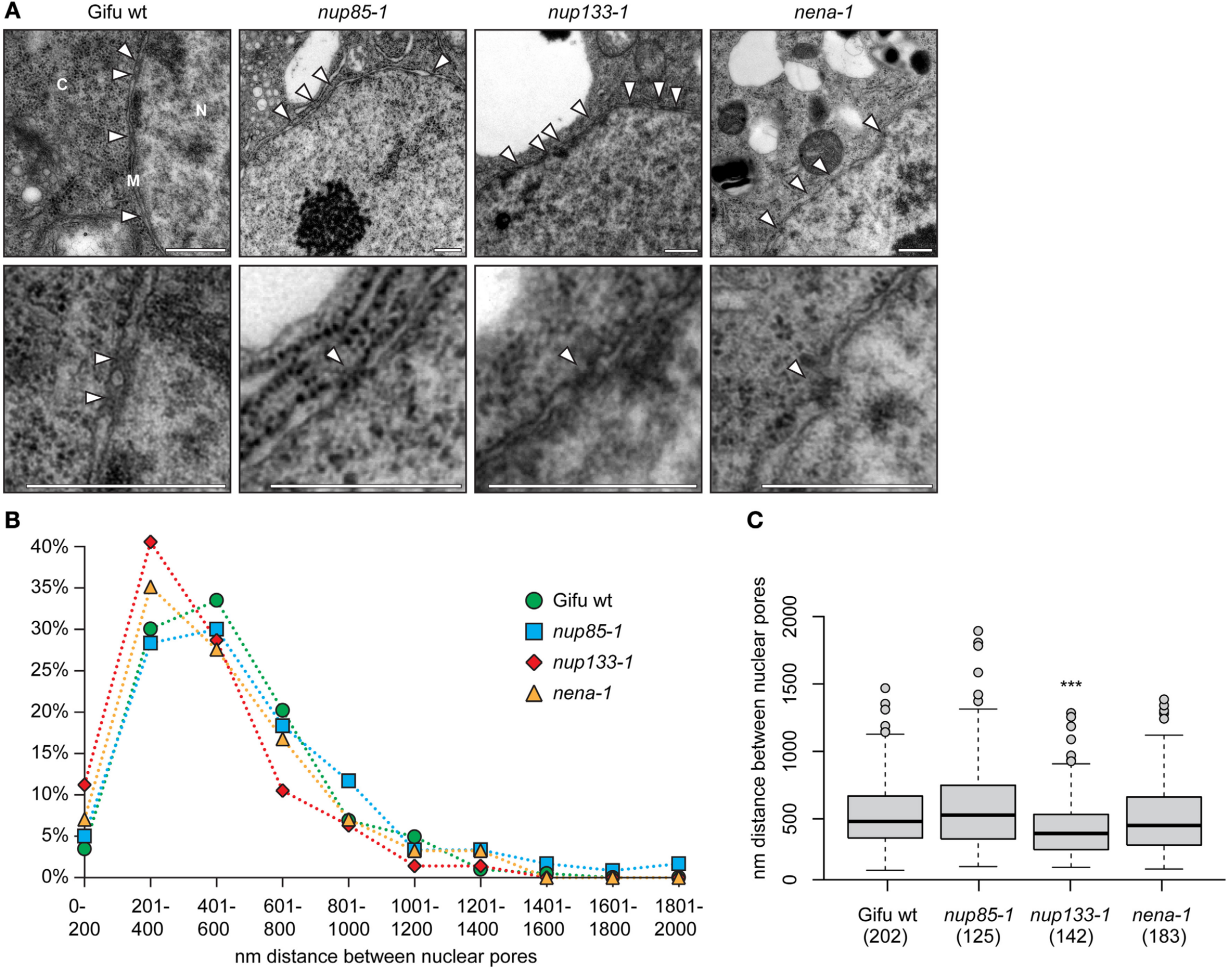
**NPC distance distribution. (A)** TEM micrographs of ultrathin sections of *L. japonicus* root tips (N, nucleus; C, cytosol; M, nuclear membranes). White arrow heads indicate the position of nuclear pores. The lower images show a magnified view of individual pores. Scale bar = 500 nm. **(B)** Distance distribution between adjacent NPCs. **(C)** Box plot showing NPC distances. “^***^” indicates significant difference from the wild type. (ANOVA revealed significant differences between average distances with *F*_(3, 648)_ = 7.63, *p* < 0.001. Dunnett test all vs. wild type on log transformed data identified *nup133-1* as having a smaller mean than wild type; *p* < 0.001). The number of measured distances for each line is indicated in brackets below the x-axis.

We compared ultrathin sections of *L. japonicus* root tips of wild type plants with those of mutant lines *nup85-1*, *nup133-1* and *nena-1* (Table 1) by transmission electron microscopy (TEM). No obvious morphological changes to the nuclear membranes or nuclear morphology could be detected beyond the intra-genotype variation (Figure 2A). NPC distribution was quantified in detail by measuring the distances between adjacent nuclear pores. The main criteria for identification of NPCs were a visible gap in the nuclear membranes, as well as a darker staining of the NPC area (Figure 2A). The overall distance distribution was comparable in mutant and wild type nuclei (Figure 2B). Average distances between pores were 533 nm for Gifu wild type, 599 nm for *nup85-1*, 440 nm for *nup133-1* and 508 nm for *nena-1*. A multiple comparisons of means (all vs. Gifu wild type) using Dunnett contrasts identified *nup133-1* as having a smaller mean than the wild type, indicating a slightly increased density of NPCs. In contrast, *nup85-1* and *nena-1* did not differ from the wild type (Figure 1C).

### Loss of *L. japonicus* NENA and NUP85 reduced the protein levels of other putative NUP107–160 subcomplex members

**Figure 3:**
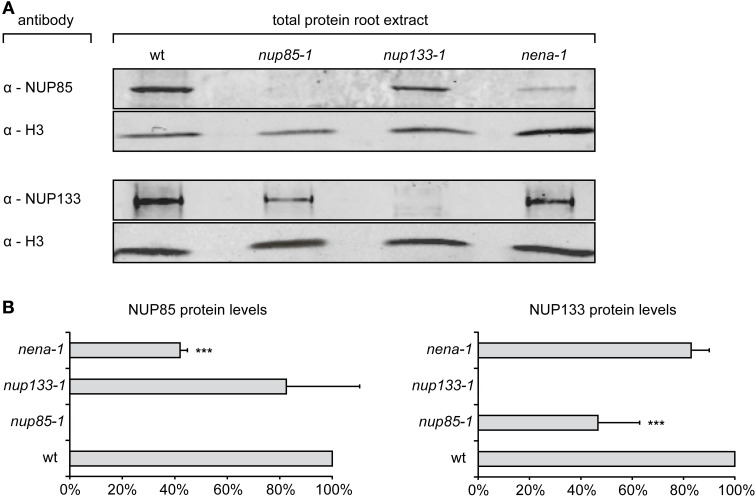
**Quantification of NUP85 and NUP133 protein levels. (A)** Total protein extract from *L. japonicus* roots was separated by SDS-PAGE, blotted on membrane and immunolabelled with α-NUP85, α-NUP133, and α-histone H3 antibodies. **(B)** For quantification, signal intensities of NUP85 and NUP133 were normalized by the histone H3 signal and shown as percentages of wild type levels (wt). Mean protein levels were averaged from three independent experiments. Error bars show SD, “^***^” indicate significant differences from the wild type (*P* ≤ 0.05, *t*-test).

Total protein extracts of Gifu wt, *nup85-1*, *nup133-1* or *nena-1* roots were probed with specific α-NUP85 or α-NUP133 antibodies and simultaneously with antibodies against histone H3. Specific NUP85 and NUP133 signals were not detected in the *nup85-1* and *nup133-1* extracts respectively, indicating that full length protein was absent from the mutants. However, the presence of residual truncated NUP85 and NUP133 protein could not be ruled out. The NUP85 antibody targets the carboxy-terminus of the protein beyond the *nup85-1* nonsense mutation site and would therefore not bind to a truncated version. The NUP133 antibody was raised against a peptide of 21 amino acids, of which 6 amino acids are affected by the frame shift mutation in *nup133-1* and might therefore also no longer detect the predicted truncated NUP133 version of 303 amino acids. In case of *nena-1* it was previously indicated that the allele is a complete null (Groth et al., 2010). For NUP quantification, three independent root extracts were assayed for wild type and each mutant line, and relative protein levels of NUP85 and NUP133 were determined via normalization against histone H3 (Figure 3). No significant change relative to wild-type was observed for either NUP133 in the *nena-1* background, or for NUP85 in the *nup133-1* background. However, protein abundance of NUP85 in the *nena-1* mutant and NUP133 in the *nup85-1* mutant was significantly reduced to about 40% and 50% of wild type levels, respectively (Figure 3). This indicated that the loss of one nuclear pore component in *L. japonicus* interfered with the protein levels of other NUP107–160 sub-complex members.

### *nup85/nup133* double mutants were severely affected in development in a temperature-dependent manner

**Figure 4:**
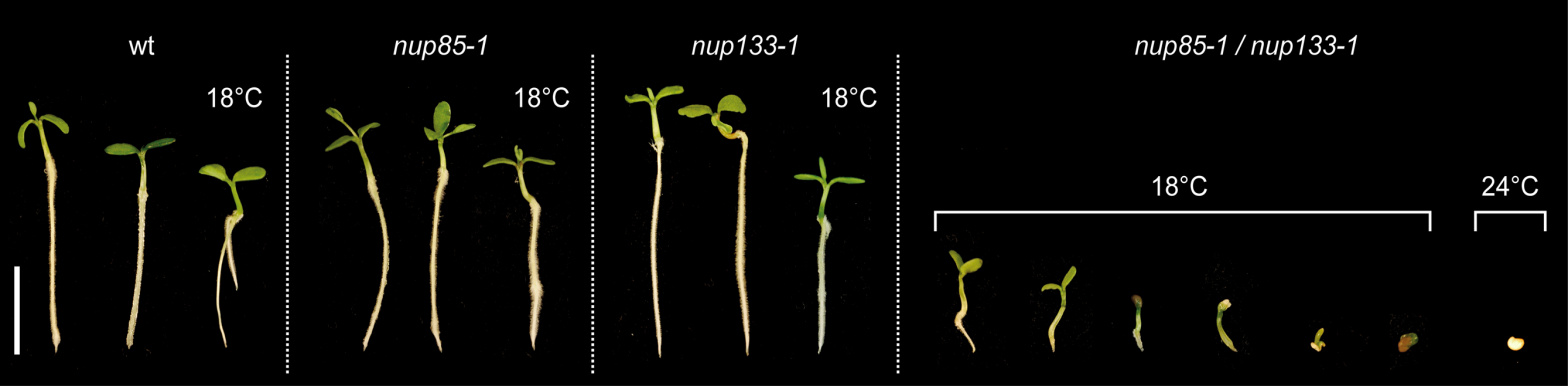
**Analysis of *nup85/nup133* double mutants**. The loss of both NUP85 and NUP133 caused severe growth and developmental defects in the double mutants. At low temperature (18°C) cotyledons and short roots could still develop, while at higher temperature (24°C), plant development was arrested at the early germination stages. Plants and undeveloped seeds were cultivated for 16 days. Scale bar = 1 cm.

To study effects associated with the complete loss of more than one nucleoporin, *nup85-1* and *nup133-1* mutants were crossed to generate double mutant lines. Individual F1 progeny heterozygous for both loci were identified by PCR based genotyping (not shown) and selfed. As both NUP85 and NUP133 loci are located on top of *L. japonicus* chromosome 1 in a genetic distance of 10 cM, only about 1% of the F2 offspring were expected to be homozygous *nup85/nup133* mutants (about 10% of the gametes should carry both *nup85-1* and *nup133-1* mutations). Since no double mutants could be obtained in 96 analyzed F2 plants, F2 individuals that were homozygous for one mutation and heterozygous for the other were selected and again selfed. In the F3 offspring of such individuals, *nup85/nup133* double mutants could be identified that showed severe and temperature-dependent developmental defects. At 24°C, double mutants were found only among imbibed seeds that did not germinate. In contrast, homozygous *nup85/nup133* plants incubated at 18°C were able to develop roots and cotyledons. However, they were severely impaired in growth compared to wild type and *nup85* or *nup133* single mutants (Figure 4). 25% of the selected F2 progeny were expected to be homozygous for both *nup85* and *nup133*, yet of the analyzed individuals only 7.7% (7 of 91) were double mutants and the observed allele distribution was significantly different from the expected values (*p* < 0.01; *X*^2^ test). Previously, a reduction in seed yield was also reported in single *nup85* and *nup133* mutants as well as shorter pollen tubes in *nup85* mutants (Kanamori et al., 2006; Saito et al., 2007). This suggests that *nup85* and *nup133* mutations affect gametophyte and seed development, resulting in fewer seeds carrying both mutations.

## Discussion

Here we studied the effects of mutations in genes coding for the *L. japonicus* NUP107–160 subcomplex members NUP85, NUP133, and NENA (SEH1). Unlike in *S. cerevisiae nup85* and *nup133*, (Doye et al., 1994; Li et al., 1995; Siniossoglou et al., 1996), *L. japonicus nup85, nup133* and *nena/seh1* mutants do not exhibit severe alterations in NPC distribution or nuclear envelope structure, except for a decrease in the average distance of neighboring NPCs in *nup133*. The three *L. japonicus nup* mutants and *A. thaliana nup133* and *seh1* are largely indistinguishable from wild type plants (Kanamori et al., 2006; Saito et al., 2007; Groth et al., 2010; Wiermer et al., 2012), while *S. cerevisiae nup85, nup133* and *seh1* mutants show severe fitness defects (Pemberton et al., 1995; Siniossoglou et al., 1996). This lack of more prominent phenotypes at the whole plant and at the nuclear level suggests that with regard to general functions the plant NPC can tolerate the loss of single structural NUPs better than the *S. cerevisiae* NPC. Phenotypes in animal *nup* mutants that are not correlated with changes in nuclear pore number are often attributed to specific functions of individual NUPs (Raices and D'Angelo, 2012). However, this is difficult to confirm, as mutations in single NUPs could affect other complex components.

Our data show that loss of *L. japonicus* NUP85 or NENA/SEH1 causes a reduction in the protein abundance of other putative NUP107–160 constituents. Similar effects have been previously observed in HeLa cells, where partial RNAi mediated knock-down of individual NUP107-160 NUPs (including NUP85, NUP133 and SEH1) lead to co-depletion of a number of other distinct NUPs from the nuclear envelope and in some cases to their proteolytic degradation (Boehmer et al., 2003; Harel et al., 2003; Walther et al., 2003; Loïodice et al., 2004; Rasala et al., 2006). The reduction of NUP85 levels in the *L. japonicus nena* mutant could be a result of the direct association of SEH1 and NUP85 (Groth et al., 2010). *In vitro* experiments previously demonstrated that yeast SEC13 can form a complex with NUP85 in the absence of SEH1, suggesting a certain level of redundancy in the architecture of the NUP84 subcomplex (Debler et al., 2008). A reduced amount of soluble NUP85-SEC13 indicated that the complex was less stable than the NUP85-SEH1 interaction (Debler et al., 2008). This destabilization could explain the decrease of NUP85 levels in the *Lotus nena-1* mutant, assuming that NENA/SEH1 was replaced by SEC13. As the NUP85-SEH1 interaction is structurally very similar to the NUP145C-SEC13 pair (Hsia et al., 2007), it is also conceivable that the loss of NENA causes a partial displacement of NUP85 by NUP145-SEC13, which in turn could lead to the increased degradation of NUP85. The fact that NUP85 levels were not reduced in the *nup133-1* mutant, but NUP133 was reduced in *nup85-1* could potentially be due to residual truncated NUP133 protein that was not detected by the antibodies. In yeast, NUP133 and NUP85 are positioned at opposite ends of the Y shaped NUP84 complex and do not directly interact (Lutzmann et al., 2002; Brohawn et al., 2008; Fernandez-Martinez et al., 2012). The reduction of NUP133 in the *nup85* mutant therefore suggests a more widespread alteration of the NUP107-160 complex. It is likely that the shared phenotypes in symbiotic signaling in *nup85*, *nup133* and *nena* are due the changes in the subcomplex and are not a function of the individual nucleoporins. In yeast mutants, NPC clustering and fitness phenotypes were shown not to correlate in severity, but instead could be mapped to different NUP84 subcomplex regions (Fernandez-Martinez et al., 2012). In the case of *L. japonicus*, it would also be interesting to analyze other NUP107-160 subcomplex mutants, with regard to both general phenotypes as well as symbiotic defects, in order to pinpoint potential distinct functional areas of the subcomplex.

There is accumulating evidence that NPCs are more heterogeneous in their composition than previously assumed (Raices and D'Angelo, 2012). In animals, several NUPs, including NUP133 are differentially expressed in different cell types and tissues (Zhang et al., 1999; Guan et al., 2000; Olsson et al., 2004; Lupu et al., 2008; Cho et al., 2009) and mutations in multiple NUPs were correlated with tissue-specific diseases (Huebner et al., 2004; Basel-Vanagaite et al., 2006; Zhang et al., 2008). Additionally over one third of all nucleoporins are present in varying stoichiometries in different cell types, including the NUP107-160 subcomplex members SEH1 and ELYS, suggesting that distinct versions of the subcomplex exist (Ori et al., 2013). This flexibility in nuclear pore composition potentially allows for stable NPC configurations even in the absence of individual NUPs, however certain functions can potentially no longer be fully performed.

The fact that *nup85/nup133* double mutants have more severe general phenotypes than either of the single mutants, highlights the functional overlap of the proteins and suggests that loss of more than one NUP107–160 complex component leads to a break-down of basal NPC functions. Stronger phenotypes have also been reported in double mutants of *A. thaliana nup96* and *nup160* (Parry et al., 2006) as well as *nup133* and *nup160* (Wiermer et al., 2012). Additionally in *S. cerevisiae* most combinations of double mutants affecting NUP107–160 complex components are synthetically lethal (Siniossoglou et al., 1996). In vitro experiments with reconstituted *Xenopus* nuclei have previously shown that depletion of NUP85 and NUP133 completely prevented the assembly of the NUP107-160 subcomplex (Harel et al., 2003). It is therefore likely that in the *L. japonicus nup85/nup133* double mutants the subcomplex, and in extension the NPC as a whole, are strongly affected. However, since cell division occurs, cellular functions seem to be maintained to some extent, making it unlikely that the NPC cannot assemble at all.

The symbiotic phenotypes in *L. japonicus nup85, nup133* and *nena* mutants (Kanamori et al., 2006; Saito et al., 2007; Groth et al., 2010) and the growth defects in several yeast *nup* mutants, including *nup133* and *nup85* (Doye et al., 1994; Li et al., 1995; Siniossoglou et al., 1996; Fernandez-Martinez et al., 2012) are less pronounced at lower temperatures. We demonstrated that a shift from 24 to 18°C also reduces the severity of the developmental defects in the Lotus *nup85/nup133* mutants. This suggests that depending on low temperature, the plant NPC can even tolerate the loss of more than one structural nuclear pore component to a certain degree. The temperature dependence both in the single and double mutants could indicate that functional redundancy is provided by an imperfect fit of alternate NUP-NUP interactions, which are destabilized at higher temperatures.

Our data supports the view of a flexible and dynamic composition of the NPC. A certain level of redundancy in the NPC architecture can potentially compensate for the loss of even structural NUPs such as NUP85, NUP133 and NENA/SEH1 with regard to many general NPC functions in particular at lower temperatures. At the same time certain pathways, such as symbiotic signaling, that may be susceptible to quantitative changes in NPC transport capabilities, can be affected by alterations in the NPC composition or by the lack of particular NPC confirmations.

### Conflict of interest statement

The authors declare that the research was conducted in the absence of any commercial or financial relationships that could be construed as a potential conflict of interest.
