# Supplementary material for: Analysis of the Lotus japonicus nuclear pore NUP107-160 subcomplex reveals pronounced structural plasticity and functional redundancy
Source: Front Plant Sci. 2014 Jan 22;4:552. doi: 10.3389/fpls.2013.00552 (PMC3897872; doi:10.3389/fpls.2013.00552)
Supplement: Supplementary file 1 [file DataSheet1.PDF]

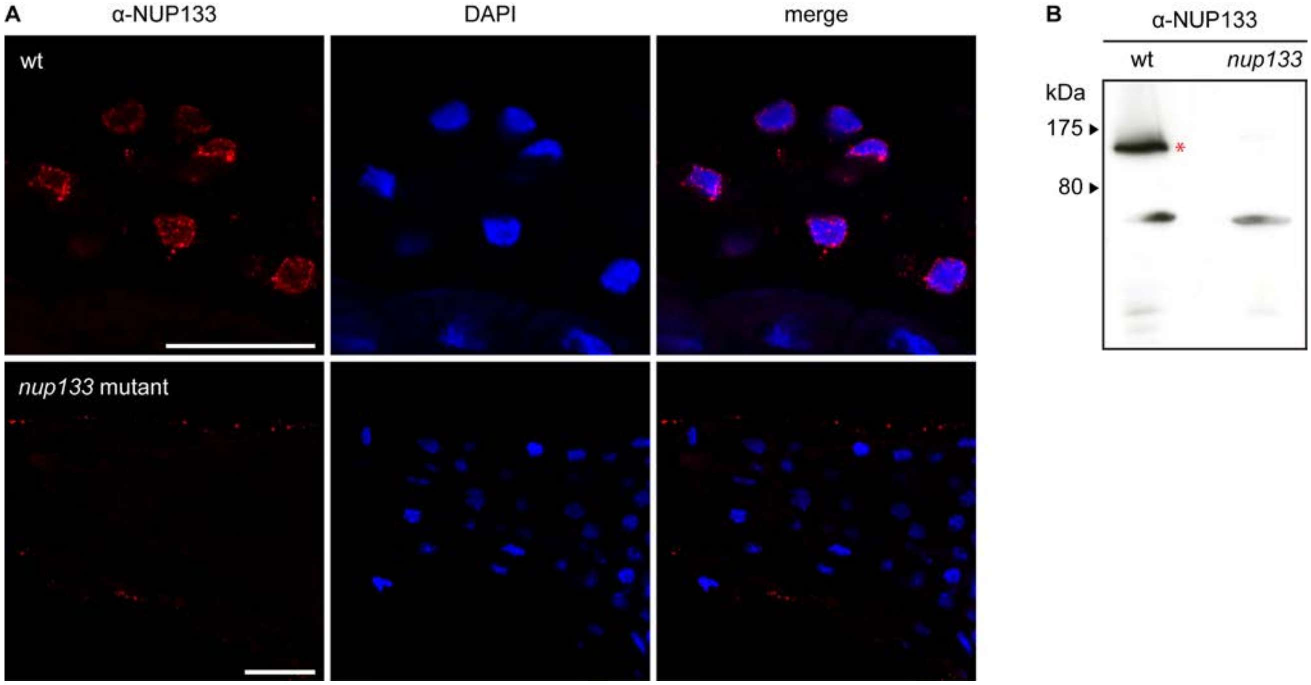

Supplementary Figure 1: Custom α-NUP133 peptide antibody specifically detected *L. japonicus* NUP133. A) NUP133 was detected in the nuclear envelope of wild type (wt) roots by whole mount immunolocalization, but not in *nup133-1* mutant roots, which showed only unspecific background signal. Secondary antibodies were coupled to Alexa647 (red). Scale bars= 25 µm. DNA was stained by DAPI (blue). B) A band corresponding to NUP133 (marked by an asterix) was specifically detected by the α-NUP133 antibody in western blot analysis. The NUP133 band was absent in the *nup133-1* mutant. A lower second unspecific band was visible both in wild type and mutant protein extracts.
